# Generation of T-cell receptors targeting a genetically stable and immunodominant cytotoxic T-lymphocyte epitope within hepatitis C virus non-structural protein 3

**DOI:** 10.1099/vir.0.037903-0

**Published:** 2012-02

**Authors:** Anna Pasetto, Lars Frelin, Anette Brass, Anila Yasmeen, Sarene Koh, Volker Lohmann, Ralf Bartenschlager, Isabelle Magalhaes, Markus Maeurer, Matti Sällberg, Margaret Chen

**Affiliations:** 1 Department of Dental Medicine, Karolinska Institutet, Stockholm, Sweden; 2 Department of Laboratory Medicine, Stockholm, Sweden; 3 Singapore Institute for Clinical Sciences, Agency for Science, Technology and Research (A*STAR), Singapore; 4 Department of Infectious Diseases, Molecular Virology, University of Heidelberg, Germany; 5 Department of Microbiology, Tumor and Cell Biology, Karolinska Institutet and Swedish Institute for Infectious Disease Control, Stockholm, Sweden

## Abstract

Hepatitis C virus (HCV) is a major cause of severe liver disease, and one major contributing factor is thought to involve a dysfunction of virus-specific T-cells. T-cell receptor (TCR) gene therapy with HCV-specific TCRs would increase the number of effector T-cells to promote virus clearance. We therefore took advantage of HLA-A2 transgenic mice to generate multiple TCR candidates against HCV using DNA vaccination followed by generation of stable T-cell–BW (T-BW) tumour hybrid cells. Using this approach, large numbers of non-structural protein 3 (NS3)-specific functional T-BW hybrids can be generated efficiently. These predominantly target the genetically stable HCV genotype 1 NS3_1073–1081_ CTL epitope, frequently associated with clearance of HCV in humans. These T-BW hybrid clones recognized the NS3_1073_ peptide with a high avidity. The hybridoma effectively recognized virus variants and targeted cells with low HLA-A2 expression, which has not been reported previously. Importantly, high-avidity murine TCRs effectively redirected human non-HCV-specific T-lymphocytes to recognize human hepatoma cells with HCV RNA replication driven by a subgenomic HCV replicon. Taken together, TCR candidates with a range of functional avidities, which can be used to study immune recognition of HCV-positive targets, have been generated. This has implications for TCR-related immunotherapy against HCV.

## Introduction

It is estimated that 180 million individuals are infected with hepatitis C virus (HCV) worldwide today, of which 130 million are chronic carriers at risk of developing liver cirrhosis and liver cancer. Being a small, enveloped RNA virus, HCV is one of the most persistent viruses in humans, and spontaneous resolution of HCV infection only occurs in a minority of the infected (Alter, 2006). Considering the steady increase of reported incidence in Europe (Rantala & van de Laar, 2008), a reduction of HCV prevalence is not anticipated in the near future.

Analyses of individuals who have resolved their HCV infection have indicated that a successful immunity requires effector T-cells. CD4^+^ and CD8^+^ lymphocyte activation early in the infection is associated strongly with eradication of HCV infection (Cucchiarini *et al.*, 2000; Diepolder *et al.*, 1996; Lechner *et al.*, 2000; Pape *et al.*, 1999; Schulze zur Wiesch *et al.*, 2005), and an early development of polyfunctional T-cells further predicts a spontaneous resolution of HCV infection (Badr *et al.*, 2008). However, failure to sustain virus-specific CD8^+^ lymphocytes is frequently observed in chronically infected patients (Lechner *et al.*, 2000). T-cells from these patients are associated with features such as functional exhaustion (Kantzanou *et al.*, 2003; Radziewicz *et al.*, 2007), developmental arrest (Cox *et al.*, 2005), tolerance induction (Cabrera *et al.*, 2004; Sugimoto *et al.*, 2003) and impairment in proliferative capacity and effector function (Wedemeyer *et al.*, 2002), which are thought to be a consequence of continued antigen stimulation and/or a viral factor related to antigen-presenting cell (Bain *et al.*, 2001; Goutagny *et al.*, 2003; Nattermann *et al.*, 2006; Ulsenheimer *et al.*, 2005; Wertheimer *et al.*, 2007).

A successful immune response in people infected with HCV is characterized by strong and persistent CTL responses. One well-defined CTL epitope is the HCV non-structural protein 3 (NS3)_1073–1081_ peptide (NS3_1073_), and responses against this epitope are often found in spontaneous resolution of HCV infection, which are well-maintained after spontaneous recovery of HCV (Wertheimer *et al.*, 2003). Several vaccines that target induction of NS3-specific T-cell immunity are currently being tested in clinical trials (Thompson & McHutchison, 2009). Recently, a novel strategy for engineering new effector T-cells against NS3_1073_ via gene transfer of a T-cell receptor (TCR) *in vitro* was described (Zhang *et al.*, 2010). Given the observation that the NS3_1073_ epitope can be associated with cross-reactivity with an influenza A virus (IAV) neuraminidase (NA) epitope, and that cross-reactive CD8^+^ T-cells have been found in fulminant acute hepatitis patients (Urbani *et al.*, 2005), access to T-cells that differ in functional avidity would be desirable for comparison.

To date, only one NS3_1073_-specific human TCR (Zhang *et al.*, 2010) has been identified and no murine alternatives are available. Murine TCRs present several advantages over human TCRs in gene modification of human T-cells, including an enhanced and more sustained level of surface expression and improved anti-tumour activity (Kieback & Uckert, 2010). Moreover, a recent murine TCR gene-therapy trial that targeted tumour antigens demonstrated a therapeutic effect in cancer patients, although some immune responses to murine TCR variable regions were found in a subset of patients (Davis *et al.*, 2010). Given these facts, we therefore chose to explore the HLA-A2 transgenic (HHD) mouse model to establish more HCV TCR gene candidates. Here a large number of functional NS3-specific T-cell–BW (T-BW) hybrid clones were obtained by fusing the BW5147 cell line and activated splenocytes cells from HHD mice immunized with an electroporation-enhanced HCV NS3 DNA vaccine. The results indicate that the T-BW hybrid clones are highly specific to the NS3_1073_ of HCV genotype 1a and 1b variants. Differences were found regarding their functional avidity and affinity to the NS3_1073_/HLA complex pentamer, as well as their ability to respond to human hepatoma cells harbouring HCV subgenomic RNA.

## Results

### Generation of HLA-A2-restricted mouse T-BW hybrid clones specific to HCV NS3

**Fig. 1.**
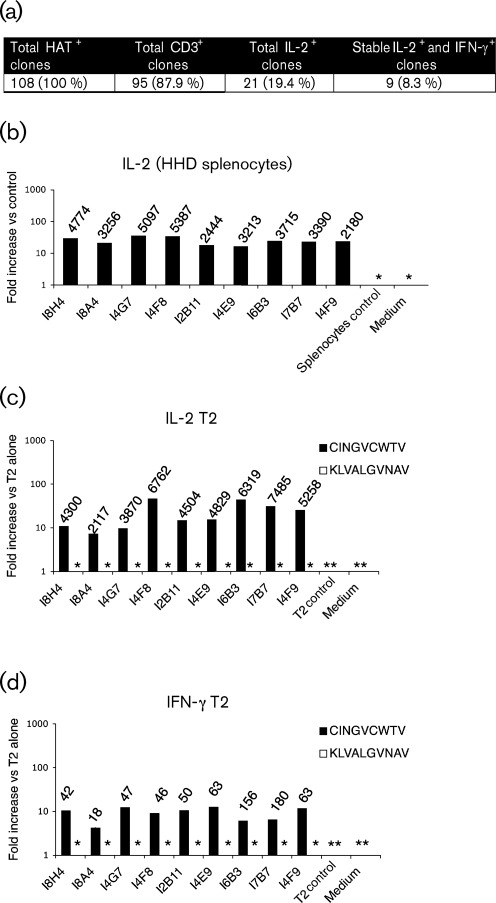
(a) Screening summary for the BW-fused HHD T-BW hybrid clones. Cytokine production against HHD or HLA-A2 target cells loaded with NS3_1073_. (b) IL-2 secretion upon stimulation with HHD splenocytes loaded with NS3_1073_. (c) IL-2 and (d) IFN-γ secretion upon stimulation with T2 cells loaded with NS3_1073_ (▪) or NS3_1406_ (□). Depicted fold induction is given as the ratio of cytokine concentration in co-cultures containing peptide-loaded target (10 µg indicated peptide ml^−1^) over the unloaded control (0 µg indicated peptide ml^−1^). Mean values of duplicate co-cultures from one experiment are shown. Amount of cytokine released (pg ml^−1^) is shown on the top of each bar. *Not detected.

We used CD8^+^ T-cells from HHD (HLA-A2 transgenic) mice (Pascolo *et al.*, 1997) immunized with the electroporation-delivered codon-optimized HCV DNA vaccine coNS3/4A-pVAX1, and performed cell fusion with BW TCR^neg^ cells. Single-cell cloning was carried out under hypoxanthine–aminopterin–thymidine (HAT) selection. T-BW hybridized clones that survived selection were screened for the surface expression of CD3 and positive clones were tested against human T2 cells (a lymphoblastic cell line) loaded with the HLA-A2-restricted NS3_1073_ (CINGVCWTV) or NS3_1406_ (KLVALGVNAV) peptide. Analysis of interleukin-2 (IL-2) and gamma interferon (IFN-γ) release in the supernatant demonstrated that nine of them recognized the NS3_1073_-loaded T2 cells and none recognized that loaded with NS3_1406_ (Fig. 1a). The reactivity was confirmed by retesting on T2 cells as well as on HHD splenocytes (Fig. 1a–d). TCR/CD3 surface expression for the respective clones was 51 % (I8H4), 81 % (I8A4), 79 % (I4G7), 85 % (I4F8), 95 % (I2B11), 95 % (I4E9), 84 % (I6B3), 52 % (I7B7) and 89 % (I4F9) at the time of the assays.

During the screening process, the hybrid clones were regularly subcloned and checked for CD3 expression to ensure that they maintained a stable CD3 expression and IL-2 activity (Supplementary Fig. S1, available in JGV Online). Although the clone I8A4 demonstrated repetitively low response against NS3_1073_, the percentage of live CD3-expressing cells and the cell-surface CD3 expression of this clone were similar to the others at the time of testing. None of the clones showed CD8 surface expression (data not shown), which is analogous to previous reported BW-derived T-cell clones (Rock *et al.*, 1990); thus, the cytokine release detected here is independent of the CD8 cofactor.

### TCR gene usage of NS3_1073_-specific T-BW cell hybrid clones

**Fig. 2.**
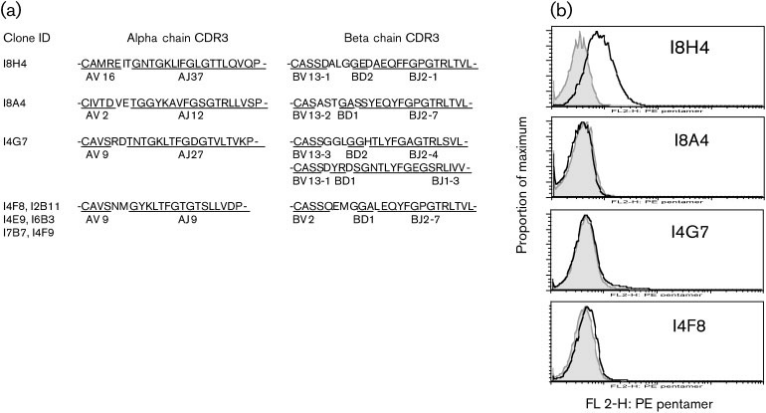
(a) Summary of TCR gene usage and CDR3 coding sequences of the nine IL-2^+^IFN-γ^+^ T-BW hybrid clones. (b) Affinity to the NS3_1073_/HLA-A2 pentamer. Indicated T-cell clones were stained with R-PE-labelled NS3_1073_/HLA-A2 pentamer (black line unfilled) or control R-PE-labelled HBV core_18–27_ pentamer (grey filled) and labelled with FITC-labelled anti-mouse CD3 antibody and analysed by FACS. Histograms of fluorescence intensity of pentamer staining were gated on the live CD3^+^ population.

Examination of the TCR usage of these T-BW hybrid clones was done in order to assess the TCR responsible for recognizing NS3_1073_. RNA was isolated from each of these clones and at least five TCR α and β gene products were sequenced. Sequence analysis showed that six of the nine clones were sister clones (Fig. 2a); AV9 and BV2 gene usage and an identical third complementarity-determining region (CDR3) sequence were found in I4F8, I2B11, I4E9, I6B3, I7B7 and I4F9. The AV9 gene was also used by I4G7, which has a unique CDR3 coding sequence using AJ27. The AV16 and AV2 genes were used by I8H4 and I8A4, respectively, and have their own CDR3 with different AJ genes. The TCR β genes were likewise determined for all clones. Three clones utilized VB13 with various subfamilies BV13-1 (I8H4), BV13-2 (I8A4), and BV13-3 and BV13-1 were found in I4G7 and they all used different BD and BJ genes. The I4F8 and its sister clones all used the BV2 gene and shared identical CDR3 nucleotide sequences.

Here, the CDR3α and CDR3β loops were between 10–13 and 11–14 aa residues, respectively. They exhibit a type II pattern bias (Turner *et al.*, 2006) that is characterized by the preference of the motifs ‘KLTFG’ (CDR3α loops) and ‘GG’ (CDR3β loops). A commonly found motif in the BJ segments here was ‘FG**P**GTR’, which is reported only in three out of 13 murine and four out of 14 human TRBJ genes (IMGT Repertoire IG and TR database; http://www.imgt.org/IMGTrepertoire/). Interestingly, the same three motifs also exist in a recently reported human TCR that is specific to the NS3_1073_ peptide (Zhang *et al.*, 2010). This result suggests that highly conserved CDR3 motifs are shared by the murine and human TCRs that are specific to the NS3_1073_ peptide.

### Affinity to NS3_1073_/HLA-A2 pentamer

Since the T-BW hybrid clones lack the CD8 coreceptor, which might be required for the stabilization of the TCR-peptide/HLA complex (Roszkowski *et al.*, 2003), we next asked whether any of these clones could bind the NS3_1073_/HLA-A2 pentamer. Following incubation with an R-phycoerythrin (R-PE)-labelled NS3_1073_/HLA-A2 pentamer, the fluorescence intensity of the staining was quantified by flow cytometry. Compared with the negative-control pentamer HBVcore_18–27_-HLA-A2, it was found that I8H4, one of the high-avidity clones, had an increased fluorescence in NS3_1073_/HLA-A2 pentamer staining (Fig. 2b).

### Comparison of the functional avidity

**Fig. 3.**
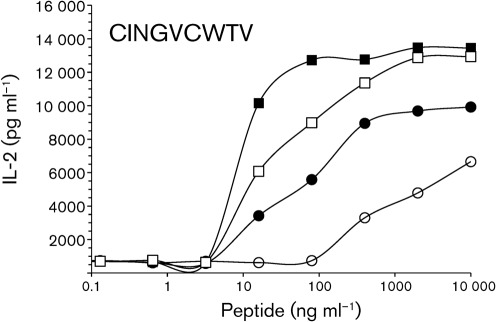
Functional avidity of T-BW hybrid clones (I8H4, •; I8A4, ○; I4G7, ▪; I4F8, □) tested against T2 cells loaded with descending concentration of NS3_1073_. Comparative results were obtained in three separate experiments.

We next asked whether the functional avidity in these T-cell hybrid clones might differ. The clones were regularly subcloned and checked by FACS and were tested when the TCR/CD3 expression was at least 50 % at the time of the experiment. They were tested with T2 target cells pulsed with diluted amounts of NS3_1073_ peptide and the results showed that, while I8A4 required at least 400 ng peptide ml^−1^ to obtain a half-maximum response of IL-2 release, about 10 ng peptide ml^−1^ was sufficient for stimulating I8H4, I4G7 and I4F8 for a half-maximum response of IL-2 release (Fig. 3). The TCR/CD3 surface expression was 51 % (I8H4), 82 % (I8A4), 88 % (I4G7) and 93 % (I4F8) at the time of this experiment. The experiment was performed three times with similar results. Based on these data, I8A4 is ranked as a low-avidity T-cell clone, while I8H4, I4G7 and I4F8 resemble moderate/high-avidity T-cells described previously (McKee *et al.*, 2005).

### Cross-genotype reactivity

**Fig. 4.**
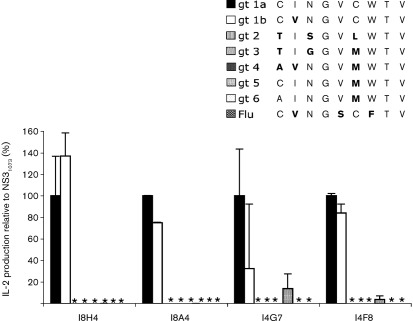
Cross-reactivity against other viral peptides that share similarity with the NS3_1073_ peptide sequence encoded by the DNA vaccine (genotype 1a). Overnight IL-2 production in T-BW hybrid clones against T2 cells loaded with the indicated viral peptide (10 µg peptide ml^−1^) was measured for each T-cell clone and is shown as a percentage of IL-2 production by genotype 1a of the NS3_1073_ peptide. Amino acids that differ from the genotype 1a are indicated in bold. Mean values and sd of triplicate co-cultures are shown. Flu, Flu-NA_231_; gt, genotype.

We next asked whether these clones cross-reacted with other viral peptides, in particular the naturally occurring genotypes of HCV NS3_1073_ and the IAV NA, Flu-NA_231_, to which cross-reactivity to HCV NS3_1073_ of genotype 1b strain has been described (Wedemeyer *et al.*, 2001). Shown in Fig. 4, we found that these T-cell clones were activated only when cultured with the genotype 1a and 1b peptide of the HCV NS3_1073_. None of the other HCV genotype peptide variants tested was sufficiently stimulatory to induce substantial IL-2 production, although all variants displayed significant binding affinity to the HLA-A2 molecule (Fytili *et al.*, 2008). No IL-2 production was found against the Flu-NA peptide (Fig. 4) or other viral peptides, including the HCV non-structural protein 5 (NS5) peptide NS5_2221_ and NS5_1992_ SPDADLIEANL and VLTDFKTWL, and human cytomegalovirus (HCMV) pp65_495_ NLVPMVATV (data not shown). The TCR/CD3 surface expression was 97 % (I8H4), 70 % (I8A4), 99 % (I4G7) and 95 % (I4F8) at the time of this experiment. The results suggest that these T-cell clones are highly specific for genotype 1 of HCV NS3.

### Relevance of each amino acid position of NS3_1073_ peptide for hybridoma reactivity

**Fig. 5.**
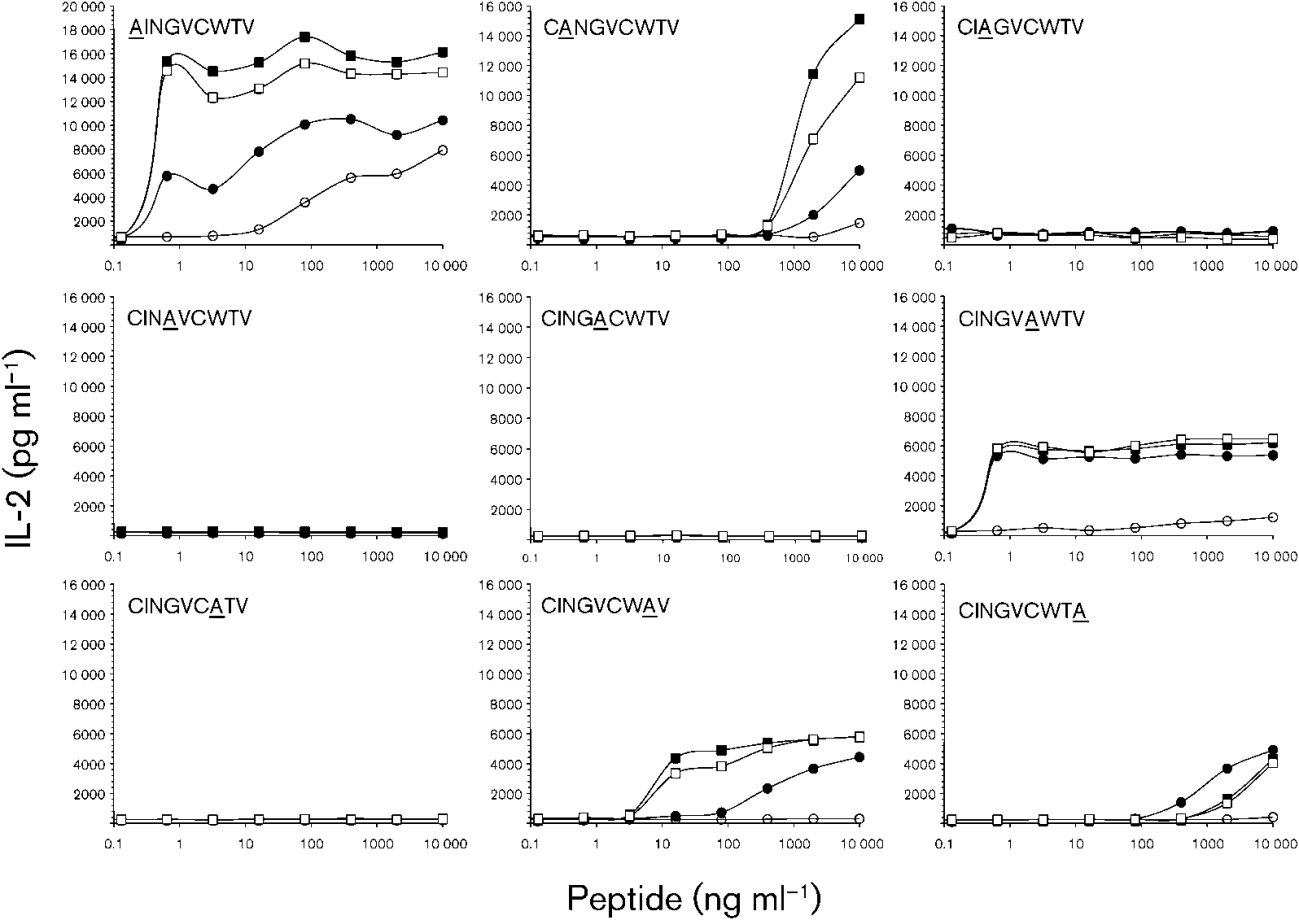
Responses to the alanine-substituted peptide analogues of NS3_1073_. Overnight IL-2 production in T-BW hybrid clones (I8H4, •; I8A4, ○; I4G7, ▪; I4F8, □) co-cultured with T2 cells loaded with descending concentrations of the alanine-substituted peptides of NS3_1073_ (substituted from positions 1 to 9). Mean values of duplicate co-cultures are shown.

Since amino acid positions 2, 7, 9 and positions 3, 4, 5 of NS3_1703_ are key positions in HLA binding and interaction with human TCR (Söderholm *et al.*, 2006), respectively, we next tested whether they have relevance for the four murine T-BW hybrid clones, 18H4, 18A4, 14G7 and 14F8. Alanine-substituted peptide analogues of NS3_1073_ loaded ono T2 cells in descending concentrations were tested against these T-cell clones. As shown in Fig. 5, alanine substitutions at position 3, 4, 5 and 7 completely abolished the IL-2 release in all four T-cell clones, which is in line with that observed for human T-cells. Complete or partial IL-2 release in response to alanine substitutions at position 1, 2, 6, 8 and 9 was, however, obtained in high-avidity clones, and was also observed at reduced peptide concentrations. This was unfortunately not observed for I8A4 (low avidity), which responded only to alanine substitution at position 1 and none of the other substitutions. The TCR/CD3 surface expression was 51 % (I8H4), 82 % (I8A4), 88 % (I4G7) and 93 % (I4F8) at the time of this experiment.

### Recognition of target cells with low HLA-A2 expression

**Fig. 6.**
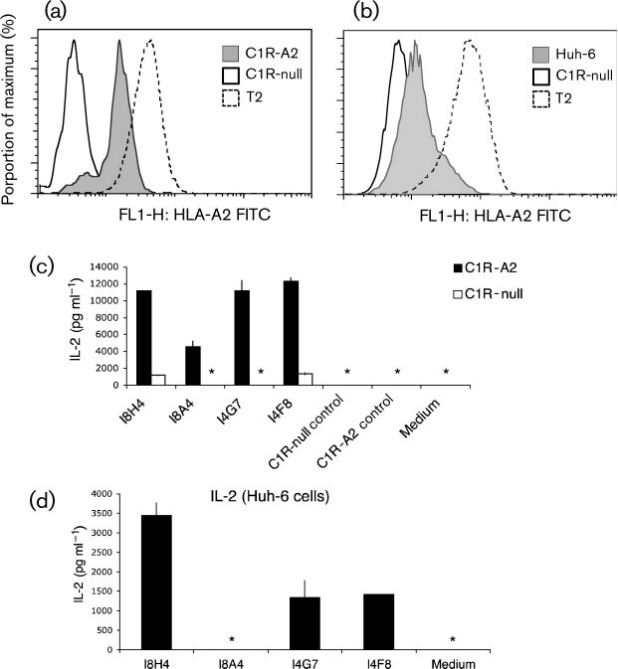
HLA-A2 expression in the B-lymphocyte C1R-A2 cell line (a, filled grey) and the hepatoblastoma Huh-6 cell line (b, filled grey) loaded with NS3_1073_ peptide compared with that of the C1R-null cells (solid line no fill) and T2 cells (dotted line). (c, d) IL-2 secretion in T-BW hybrid clones following co-culture with (c) C1R-A2 cells (▪) or C1R-null (□) cells or (d) Huh-6 cells loaded with 10 µg NS3_1073_ peptide ml^−1^. Mean values and sd of duplicate co-cultures are shown. Similar results were obtained in two separate experiments.

T2 cells are a lymphoblastic cell line commonly used as antigen-presenting cells for loading exogenous peptide and, due to the constitutive HLA-A.2 expression and TAP (transporter associated with antigen processing) deficiency, the density of these target molecules is often saturated. We next asked whether our T-cell hybrid clones recognize target cells that have low HLA-A2 expression. The hepatoblastoma Huh-6 and C1R-A2 cell lines were chosen for this experiment. Cell-surface expression of HLA-A2 assessed by staining with an anti-HLA-A2 mAb (Fig. 6a) after NS3_1073_ peptide stabilization showed that both cell lines have lower HLA-A2 expression than T2 cells. Huh-6 cells showed the lowest HLA-A2 expression, as only a fraction of the cells were positive for staining compared with the negative-control C1R-null cell line (Fig. 6b). We then assessed IL-2 production in the T-cell clones co-cultured with these target cells. Shown in Fig. 6(c), NS3_1073_ peptide-loaded C1R-A2 cells could stimulate significant IL-2 production in all four T-cell lines, at a similar magnitude to that observed for the T2 target cells. Significant IL-2 release against the peptide-loaded Huh-6 cells was, however, only detected in the high-avidity clones, especially the I8H4 clone (Fig. 6d). TCR/CD3 surface expression was 90 % (I8H4), 80 % (I8A4), 89 % (I4G7) and 90 % (I4F8) at the time of this experiment.

### Reactivity against HCV RNA replicon hepatoma cells

**Fig. 7.**
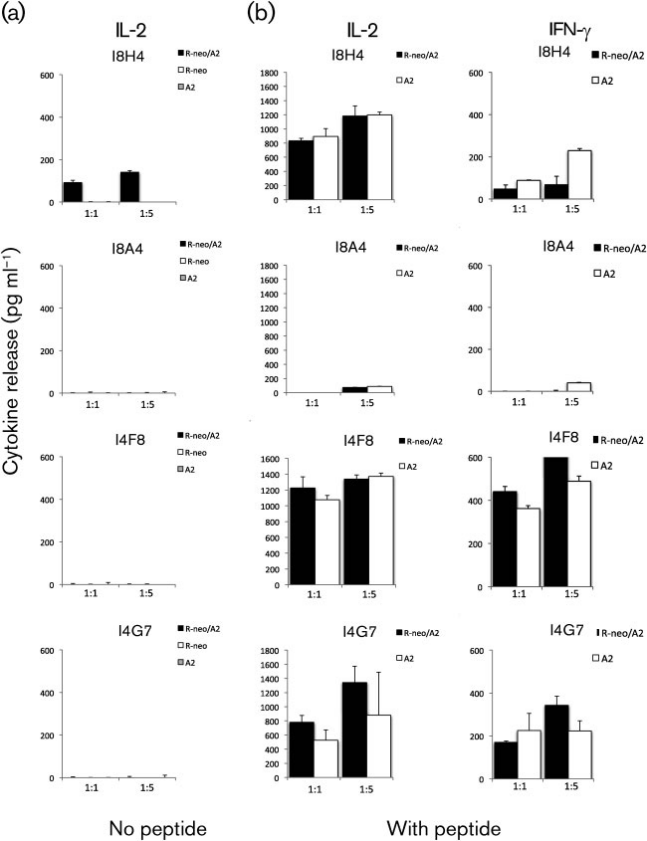
Cytokine release against the Huh-7/Lunet-derived HCV replicon cells. (a) IL-2 concentration in T-BW hybrid clone I8H4, I8A4, I4F8 or I4G7 co-cultured with the Lunet-HlaA2-neoET (R-neo/A2) replicon cells that harbour both the HCV Con1-ET subgenomic replicon and HLA-A2, or co-cultured with control cell lines Lunet-blr/neo ET (R-neo) or Lunet-HlaA2 neo (A2) that harbour only the HCV replicon or HLA-A2, respectively. (b) IL-2 and IFN-γ concentration in the T-BW hybrid clones co-cultured with Lunet-HlaA2-neoET (R-neo/A2) HCV replicon cells, or the control Lunet-HlaA2 neo (A2) cells that express HLA-A2 but no HCV replicon, that were loaded with NS3_1073_ peptide (genotype 1a, 10 µg ml^−1^). Co-cultures consisted of 1×10^5^ of indicated Lunet cells and T-BW cells in the ratio 1 : 1 or 1 : 5. Mean values and sd of duplicate co-cultures are shown.

We next analysed the ability of the T-cell hybrids to recognize the Huh-7/Lunet HCV replicon cells that harbour the subgenomic HCV replicon. Huh-7/Lunet hepatoma replicon cells maintain constant levels of HCV replication over several years and represent an excellent model of persistent HCV infection (Bartenschlager & Sparacio, 2007). As Huh-7 cells lack HLA-A2 expression, they are stably transduced with lentiviral vectors expressing HLA-A2 and a selectable marker encoding the blasticidin-resistance gene, and transfected with the HCV genotype 1b Con1-ET subgenomic replicon and a selection marker conferring the neomycin-resistance gene by using an approach described previously (Ahlén *et al.*, 2007). As shown in Fig. 7(b), antigen-specific IL-2 production by the T-cell clones was detected after co-culture at different ratios with peptide (NS3_1073_ genotype 1a)-loaded HCV replicon cells harbouring both the Con1-ET replicon and HLA-A2 (R-neo/A2) or control cells expressing only the HLA-A2 (A2). In these cultures both IL-2 and IFN-γ were detected, particularly from the high-avidity I8H4, I4F8 and I4G7 T-cell clones, and this indicates that the lentiviral-transferred HLA expression is functional. The TCR/CD3 surface expression was 90 % (I8H4), 80 % (I8A4), 89 % (I4G7) and 90 % (I4F8) at the time of the experiment. In the same experiment, we investigated whether these T-cell clones recognized the replicon cells directly without addition of exogenous peptide. Shown in Fig. 7(a), IL-2 production was detected in the co-culture of the I8H4 and HLA-A2-positive HCV replicon cells (R-neo/A2), and this IL-2 production was dependent on the ratio of the added cells. No IFN-γ was detected (data not shown). Stimulation with HCV replicon cells without HLA-A2 expression or cells with HLA-A2 expression only did not stimulated any IL-2 release from the I8H4. This result concurs with previous results that I8H4 is the only clone with affinity to the NS3_1073_/HLA-A2 pentamer (Fig. 2) and has an increased response to genotype 1b sequence (Fig. 4) and the Huh-6 target cells (Fig. 6).

### Transfer of TCR genes into naïve non-HCV specific human T-cells

**Fig. 8.**
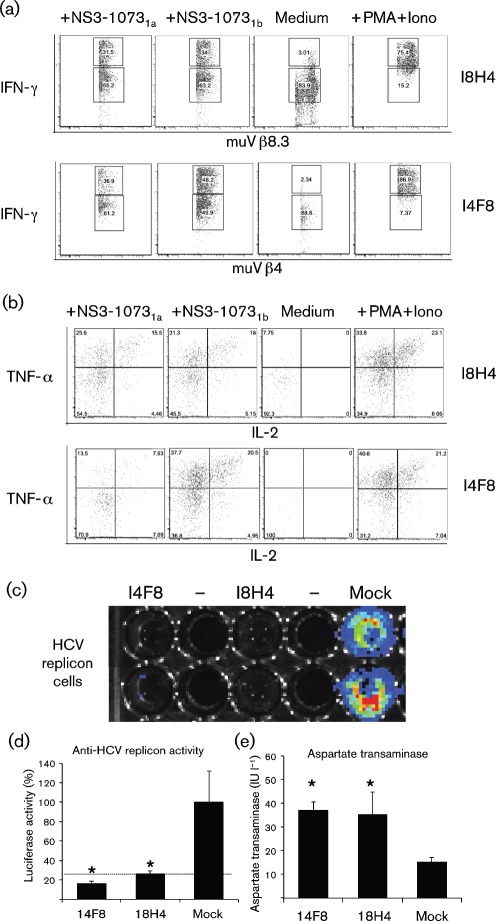
Multiple effector functions in the TCR-expressing naïve HCV-non-specific CD8 T-cells co-cultured with peptide-loaded T2 cells or the endogenous viral peptide in HCV Con1 genotype 1b replicon cells. (a) Intracellular IFN-γ staining on mouse (mu)Vβ^+^humanCD8^+^ lymphocytes in transduced T-cells co-cultured overnight with peptide (NS3_1073_ 1a or 1b)-loaded T2 cells or controls, and (b) intracellular TNF-α and IL-2 staining in the IFN-γ^+^ population in (a). (c) Bioluminescence of HCV replicon-encoded luciferase activity in Lunet-HlaA2-Luc-ubi-neo Con1 replicon cells that have been co-incubated with human T-cells transduced with I4F8 or I8H4 TCR, as assessment of antiviral inhibition specific to the endogenous NS3_1073_ viral peptide (1b). (d) Anti-HCV effect on Lunet-HlaA2-Luc-ubi-neo Con1 replicon cells, and (e) hepatocellular injury caused by TCR-transduced T-cells in quadruple co-cultures. Mean values and sd are given and expressed as percentage relative light units for luciferase (where mock corresponds to 100 %), and international units (I.U.) per litre for aspartate transaminase. * Indicates *P* value <0.01 (Student’s *t*-test) compared with mock. Dotted line indicates the cut-off value of background bioluminescence in empty wells.

Although BW hybridomas are a good model to assess TCR functions, they have a limited clinical application in humans. To know whether murine TCRs identified here are functional to redirect non-HCV-specific human T-lymphocytes against HCV^+^ cells, peripheral blood lymphocytes (PBLs) from healthy human donors were thus transduced with retroviral vectors packaged with genes encoding I8H4 TCR and the I4F8 TCR, which reacted strongly against the NS3_1073_ peptide in previous experiments. Surface expression of the respective TCRs following retroviral transduction was detected following transduction in 10–20 % CD3^+^ T-cells and not noticeably in mock, untransduced PBL controls (<0.15 %) stained with the mouse Vβ antibodies. Transduced T-cells, here defined as mouseVb^+^humanCD8^+^-expressing cells, were found to produce significant amounts of IFN-γ when stimulated with NS3_1073_ 1a or 1b peptide-loaded T2 cells (Fig. 8a). Tumour necrosis factor alpha (TNF-α) was found frequently in this IFN-γ^+^ population and a ‘triple’-positive population (IL-2^+^TNF-α^+^ IFN-γ^+^) was clearly demonstrated (Fig. 8b). The CTL function was studied on luciferase-producing bioluminescent Huh-7/Lunet HCV replicon cells (HLA-A2^+^ Luc-ubi-neo Con1^+^) that had been co-incubated with transduced T-cells using a charge-coupled device (CCD) camera. Images were taken at 20 h after co-incubation and analysed by Living Image software (version 4.2; Caliper Life Sciences). The analysis showed an efficient elimination of bioluminescent HCV replicon cells that have been co-incubated with I4F8 and I8H4 TCR-transduced T-cells (Fig. 8c, d), and a significant increase of hepatocellular aspartate transaminase was also detected in these co-culture supernatants when compared with those co-incubated with mock-transduced T-cells (Fig. 8e). In conclusion, our data indicate that murine TCRs identified by the current approach can provide antigen specificity to naïve non-HCV-specific T-cells to generate new effector T-cells that are polyfunctional and can eliminate HCV RNA-replicating hepatoma cells.

## Discussion

In this study, we describe identification of new HCV-specific T-cell clones in the HLA-A2 transgenic mouse model generated by DNA vaccination by electroporation and stable immortalization of these T-cell clones via somatic-cell hybridization with the BW5147 cell line. These T-cell clones recognize the NS3_1073_ epitope, which is one of the most important CTL epitopes in hepatitis C often associated with spontaneous resolution of HCV infection (Wertheimer *et al.*, 2003). As shown here, they are highly specific to NS3_1073_ and cross-recognition is restricted within genotype 1a and 1b virus variants and not to other related viral sequences tested. This is interesting because genotypes 1a and 1b viruses are found in up to 70 % of infected patients in the USA and Europe. Moreover, these clones differ from each other with regard to their functional avidity and affinity. This resembles the discrepancy in T-cell avidity observed in humans between donors who have recovered from HCV infection and chronically infected patients (Neveu *et al.*, 2008). In this regard, the high-avidity clones I8H4, I4G7 and I4F8 generated in this study have similar EM_50_ (effective molarity) values (approx. 10 ng ml^−1^) to those found in CD8^+^ T-cells in individuals who have recovered from HCV infection, while clone I8A4, which has a 10–100-fold-higher EM_50_ value (400 ng ml^−1^), resembles the T-cells found in chronically infected patients (Neveu *et al.*, 2008). Furthermore, there has been an elegant study by Kasprowicz *et al*. (2008) in which it was demonstrated that NS3_1073_-specific T-cells that are CD8-independent might represent cells of higher functions, i.e. require substantially less viral peptide as well as fewer cells to achieve the effector function. This might be of relevance for the mouse TCRs reported here, as they were raised in the absence of human CD8 and are capable of recognizing the target cells in a CD8-independent manner.

To our knowledge, this is the first study that has examined the TCR repertoire that is induced by an HCV vaccine. The TCR repertoire induced by natural infection of HCV has so far described the β chain (Miles *et al.*, 2011), but it is interesting to find that our murine TCRs not only share similar CDR3 lengths to those found there, but also the same sequence motifs found in a human NS3_1073_ TCR (Zhang *et al.*, 2010). Given the conserved nature of these motifs, it is likely that they play a key role in the interface between TCR and antigen contacts, tickling the T-cell to respond.

Moreover, our report is the first to demonstrate that HCV-specific T-cell hybridomas of different functional avidities towards a human HCV CTL target can be generated rapidly by DNA vaccination of HHD mice. Studies of HCV antigen presentation often require substantial amounts of HCV-specific lymphocytes/primary human T-cells (Accapezzato *et al.*, 2005; Barth *et al.*, 2005, 2008; Lapenta *et al.*, 2006) but, because of the short lifespan and the requirement of repetitive antigen stimulation with primary T-cell lines, an alternative approach to obtaining large amounts of HCV-specific T-cells would be of interest. The T-cell hybrid clones presented here could be useful reporter T-cells with mouse cytokines as reporter protein; unlike primary T-cell clones, they do not require maintenance through antigen stimulation or cytokine growth factors, but grow vigorously in simple cell-culture medium without special supplements. Their activity does not fluctuate in a cyclical manner, as occurs with some antigen-stimulated T-cell clones. Freezing and recovery are easy and, moreover, good viability and activity are usually observed. Some limitations need to be considered here. One is that avidity is determined in peptide-titration assays and not by the off-rate of pentamers after binding to the TCR. The second is that BW-derived hybrids lack CTL function because CD8 expression is suppressed (Rock *et al.*, 1990).

Despite the fact that only one hybridoma (I8H4) here recognized endogenously processed antigen from human hepatoma cells that replicate HCV RNA, we note that functional properties were further improved when I8H4 and I4F8, the latter also a high-avidity TCR, were genetically transferred and expressed on naïve non-HCV-specific human peripheral T-lymphocytes. Both murine TCRs were highly functional in human CD8 T-cells to give rise to antigen-specific polyfunctional human CD8^+^ T-cells capable of demonstrating at least four effector functions, including eliminating HCV genotype 1b replicon cells and causing aspartate aminotransferase (AST) enzyme release. This is encouraging, as polyfunctional effector T-cells capable of IL-2 production are associated with effective control of HCV (Ciuffreda *et al.*, 2008) and represent an important feature in protective T-cell memory induced by highly efficacious human vaccines (Ahmed & Akondy, 2011). Because HCV genotype 1 is in general associated with drug resistance, the notion that our TCRs can cross-recognize genotype 1a and genotype 1b viral peptides makes them interesting as immunotherapy candidates.

It is true that we have failed to induce NS3_1406_ T-cells in this study, because in HLA transgenic mice they are better induced by peptide vaccination (Brinster *et al.*, 2001). However, with the current approach we have now generated new TCRs that target NS5 CTL epitopes that are associated with control of acute self-limited hepatitis C (A. Pasetto and others, unpublished data). With the bioluminescent HCV replicon cells as an indicator for CTL efficiency, it would be interesting to compare the antiviral potentials in TCR-modified effector T-cells with various peptide specificities.

In summary, the current study has demonstrated an efficient way to generate TCR candidates that range in different functional avidities, with specificity against an important HCV CTL target. It has implications on the development of HCV immunotherapy and the understanding of T-cell recognition of HCV-infected cells.

## Methods

### 

#### Animals.

Inbred HHD-C57BL/6 (HHD^+^H-2D^b−/−^β2m^−/−^) mice transgenic for HLA-A2.1, and deficient for both H-2D^b^ and murine β2-microglobulin (β2m) (kindly provided by Dr F. Lemonnier, Institut Pasteur, France) (Pascolo *et al.*, 1997) were maintained at the Karolinska Institutet, Division of Comparative Medicine (AKM), Clinical Research Centre, Karolinska University Hospital The regulations of the Ethical Committee for animal research at the Karolinska Institutet were followed.

#### Human PBMCs.

PBMCs from healthy blood donors were collected at the Karolinska University Hospital under informed consent, and isolated using Ficoll-Hypaque density-gradient centrifugation. Ethical permission was obtained from the Regional Ethical Review Board (EPN) of the Karolinska Institutet.

#### Plasmids and synthetic peptides.

The plasmid coNS3/4A-pVAX1, containing the full-length codon-optimized NS3/4A gene of HCV genotype 1a, has been described previously (Frelin *et al.*, 2003, 2004). The HCV NS3 genotype 1a peptides CINGVCWTV (aa 1073–1081) and KLVALGVNAV (aa 1406–1415) are referred to as NS3_1073_ and NS3_1406,_ respectively (Brinster *et al.*, 2001; Himoudi *et al.*, 2002). Mutant peptides of NS3_1073_ with indicated alanine (Ala) substitutions were used as indicated. Other viral peptides tested were: Flu-NA CVNGSCFTV (aa 231–239); HCV NS3_1073_ genotype 1a CINGVCWTV, genotype 1b CVNGVCWTV, genotype 2 TISGVLWTV, genotype 3 TIGGVMWTV, genotype 4 AVNGVMWTV, genotype 5 AINGVMWTV; HCV NS5_2221_ and NS5_1992_ SPDADLIEANL (aa 2221–2231) and VLTDFKTWL (aa 1992–2000); and HCMV pp65 NLVPMVATV (aa 495–504). All peptides were synthesized (purity >70 %) by ChronTech Pharma AB and EZBiolab.

#### Cell lines.

BW5147 alpha-beta-cell line (BW TCR^neg^ cells; kindly provided by Drs J. Kappler and P. Marrack at the National Jewish Medical and Research Center) were grown in complete BW medium [Dulbecco’s modified Eagle’s medium (DMEM) 10 % FBS supplemented with non-essential amino acids, l-glutamine and gentamicin]. T2, C1R-A2 and C1R-null cells were grown in RPMI 10 % FBS with 2 mM l-glutamine and 100 mM HEPES. The hepatoblastoma Huh-6 cell line was cultured in DMEM with 10 % FBS. T2 is an HLA A2.1^+^ cell line, a cloned hybrid between the 721.174 (variant of the LCL 721 B-lymphoblastic cell line) and CEMR.3 (8-azaguanine- and ouabain-resistant clone of the CEM T-lymphoblastic cell line). C1R-A2 and C1R-null are Epstein–Barr virus--transformed B-lymphocyte cell lines. Endogenous peptides bound to HLA-A2 molecules on C1R-A2 cells were removed by mild acid treatment (pH 3.3) and replaced by HCV NS3-specific HLA-A2 peptides (Jäger *et al.*, 1999). All media and supplements were purchased from Invitrogen.

#### Generation of HCV NS3-specific T-BW hybridoma clones.

Splenocytes and lymph node cells were isolated from coNS3/4A-DNA immunized HHD-C57BL/6 mice as described previously (Ahlén *et al.*, 2007). Following 5 days stimulation with HCV NS3 peptides, CD8^+^ T-cells were purified using CD8 MACS MicroBeads (Miltenyi Biotec) and fused to BW TCR^neg^ cells. Briefly, the CD8^+^ cells were mixed with BW TCR^neg^ cells in a 1 : 5 ratio and PEG1450 (Sigma) was added; thereafter, the cells were resuspended in minimal essential medium (MEM) and incubated for 5 min at 37 °C, and cultured for 48 h before selection in HAT (Sigma Aldrich) and HT (HAT without aminopterin; Gibco, Invitrogen) media. Hybridized cell clones were maintained in complete BW medium. Positive clones were retested and regularly subcloned and checked for CD3 expression.

#### HCV replicon cells.

Hepatoma Huh-7-Lunet cells designated Lunet-HlaA2-neoET, Lunet-blr/neo ET or Lunet-HlaA2 (neo) or Lunet-HLA-A2-Luc-ubi-neo Con1 were generated by rotocol similar to that described previously (Ahlén *et al.*, 2007). The Lunet-HlaA2-neoET has ectopic HLA-A2 expression and a selectable HCV subgenomic RNA replicon of genotype 1b, harbouring replication-enhancing mutations in NS3 and NS4B (Con1-ET). This is the same for Lunet-HLA-A2-Luc-ubi-neo Con1, but it also co-expresses the firefly luciferase gene. The control replicon cell line Lunet-blr/neo ET was transduced with an empty viral vector without the HLA-A2 gene. These cells were maintained in complete DMEM with addition of blasticidin S hydrochloride (3 µg ml^−1^) and G418 (1 mg ml^−1^). Lunet-HlaA2, the other control cell line (expressing HLA-A2 under blasticidin selection but without the HCV replicon), was maintained in the same DMEM as above but with 3 µg blasticidin S hydrochloride ml^−1^ and no G418. For the co-culture experiments, the Lunet cells were washed and reseeded 1 day before in antibiotic-free medium. All medium and supplements were purchased from Invitrogen.

#### Antibodies and flow cytometry.

Antibodies against mouse CD3, CD8 and human HLA-A2 (BD Biosciences), and R-PE-labelled HLA-A*0201 Pro5 pentamers refolded with HCV NS3_1073_ or HBV core_18–27_ (ProImmune) were used. For intracellular multicolour FACS staining, TCR-transduced PBMCs were incubated overnight with indicated stimuli. GolgiPlug (BD Biosciences) was added during the final 12 h. PMA at 50 ng ml^−1^ and ionomycin at 500 ng ml^−1^ (Sigma) were used as a positive control. Cells were then washed and stained with: Pacific Blue–anti-human CD3 (Biolegend), allophycocyanin–Cy7–anti-human CD8a (Biolegend), FITC-labelled anti-mouse Vβ 8.3 (BD Biosciences), anti-mouse Vβ8.1-8.2 (BD Biosciences), anti-mouse Vβ6 (BD Biosciences), anti-mouse Vβ4 (BD Biosciences), allophycocyanin–anti-human IL-2 (Biolegend), PE–anti-human IFN-γ (Biolegend) and PE–Cy7–anti-human TNF (BD Biosciences). A BD Cytofix/Cytoperm fixation/permeabilization kit was used. Cells were analysed using a BD LSRFortessa flow cytometer and FlowJo 9.2 (Tree Star) software.

#### Measurement of cytokine release.

Target cells (2×10^5^) were cultured for 24 or 48 h with equal numbers or the indicated number of each BW-T cell hybrid clone in duplicates with or without different viral peptides. Concentrations of mouse IL-2 and IFN-γ in the supernatant were measured using mouse IL-2 or IFN-γ ELISA **(**Mabtech) and calculated against standard curves generated with a cytokine standard.

#### TCR gene typing and sequencing, and retroviral TCR constructs.

Total RNA was reverse-transcribed to cDNA using SuperScipt III RT enzyme (Invitrogen). TCR variable alpha (VA) and variable beta (VB) chain typing was done by PCR with Platinum *Taq* polymerase (Invitrogen) and primer sets covering the entire murine TCR VA and VB repertoire. PCR products were cloned (pCR-4 TOPO system, Invitrogen) and the plasmids were sequenced by Eurofins MWG (Ebersberg). Sequences were analysed and classified according to the nomenclature given in the Immunogenetics database (European Bioinformatics Institute, Cambridge, UK). Full-length TCR genes were amplified and, after sequence confirmation, synthetic genes linked with the autoprotease 2A sequence were made (GeneArt, LifeTechnology) and assembled into pMP-71-G-Pre retroviral plasmid (kindly provided by Wolfgang Uckert, Max-Delbrück-Center for Molecular Medicine). Phoenix amphotropic packaging line (Nolan’s lab, Standford University) was used to package the expression plasmids for expression in primary human T-cells. Calcium phosphate transfection was done with 20 µg of each vector (pMP71-NS3-H4, pMP71-NS3-F8, pMP71-NS5-19, pMP71-NS5-69 and pMP71-EGFP) and 12.5 µl of 50 mM chloroquine.

#### Retrovirus transduction.

Human PBMCs were stimulated with 600 or 300 U IL-2 ml^−1^ (R&D System or Prepotech) and 50 ng anti-CD3 ml^−1^ (OKT-3 eBioscience). Lymphocytes were harvested and transduced by spinoculation on retronectin-coated wells with polybrene (Millipore). Spinoculation was repeated the next day and TCR surface expression was analysed by FACS 72 h after the first spinoculation.

#### Bioluminescence cell imaging and transaminase measurement.

Either 50 000 or 100 000 Lunet-HlaA2-Luc-ubi-neo Con1 cells were co-cultured with transduced or mock-transduced T-cells in a ratio of 2 : 1. Following 20 h co-incubation the medium was replaced with luciferin solution prior to imaging with a charge-coupled device camera. Signals from bioluminescent Lunet-HlaA2-Luc-ubi-neo Con1 cells were analysed with the Living Image Software version 4.2 and IVIS Spectrum instrument (Caliper Life Sciences). The AST level in supernatants was quantified by a validated AST assay at the Clinical Chemistry Laboratory at the Karolinska University Hospital.
